# Methodological challenges in the comparison of infant fMRI across age groups

**DOI:** 10.1016/j.dcn.2017.11.003

**Published:** 2017-11-11

**Authors:** Rhodri Cusack, Olivia McCuaig, Annika C. Linke

**Affiliations:** a Brain and Mind Institute, Western University, Canada; b Trinity College, Dublin, Ireland; c San Diego State University, USA

**Keywords:** fMRI, fcMRI, Functional connectivity, Infants, Neonates, Preterm

## Abstract

Functional MRI (fMRI) in infants is rapidly growing and providing fundamental insights into the origins of brain functions. Comparing brain development at different ages is particularly powerful, but there are a number of methodological challenges that must be addressed if confounds are to be avoided. With development, brains change in composition in a way that alters their tissue contrast, and in size, shape, and gyrification, requiring careful image processing strategies and age-specific standard templates. The hemodynamic response and other aspects of physiology change with age, requiring careful paradigm design and analysis methods. Infants move more, particularly around the second year of age, and move in a different way to adults. This movement can lead to distortion in fMRI images, and requires tailored techniques during acquisition and post-processing. Infants have different sleep patterns, and their sensory periphery is changing macroscopically and in its neural pathways. Finally, once data have been acquired and analyzed, there are important considerations during mapping of brain processes and cognitive functions across age groups. In summary, new methods are critical to the comparison across age groups, and key to maximizing the rate at which infant fMRI can provide insight into the fascinating questions about the origin of cognition.

Functional magnetic resonance imaging (fMRI) in infants is providing a new window onto the emergence of cognitive functions such as auditory-language processes (Dehaene-Lambertz et al., 2006, Dehaene-Lambertz et al., 2002; Wild et al., 2017; Perani et al., 2010, Anderson et al., 2001), visual processes (Biagi et al., 2015, Altman and Bernal, 2001, Lee et al., 2012, Deen et al., 2017), and somatosensory-motor processes (Allievi et al., 2015, Arichi et al., 2010). Neural measures have particular value in young infants, as characterizing cognitive functions from behavior alone before language fluency develops remains difficult, despite a great deal of ingenuity in paradigm design in the field of developmental psychology (Cusack et al., 2016). Infant fMRI can also address a pressing clinical need, by providing a new way to detect atypical neurocognitive development following brain injury, which will facilitate earlier and more effective intervention (Smyser et al., 2013, Tusor et al., 2013). Pre-existing methods that rely upon measures of brain structure such as anatomical MRI or cranial ultrasound are only moderately predictive of atypical development, as the infant brain has enormous plasticity and can reorganize function even in the presence of substantial structural injury. Measures of brain function, therefore have the potential to provide valuable additional information (Herzmann et al., 2017; Linke et al., 2017).

Key to understanding brain development with infant fMRI is contrasting of different age groups in longitudinal or cross-sectional comparisons. This can be done between infants of different ages, between preterm and term groups, or between infants and adults (see Fig. 1 for an overview of previous studies). For this review, we consider activation fMRI, the measurement of brain activation in response to a stimulus, and functional-connectivity fMRI, the analysis of the connectivity between brain regions as assessed through the degree of synchronous fluctuation in brain activity. There are many methodological challenges in comparing fMRI measurements across age groups. The goal of this narrative review is to identify those challenges, to facilitate the appropriate caution when interpreting the literature and to facilitate the design of future studies. We survey challenges that affect the acquisition, analysis, and interpretation of fMRI comparisons between age groups.

**Fig. 1:**
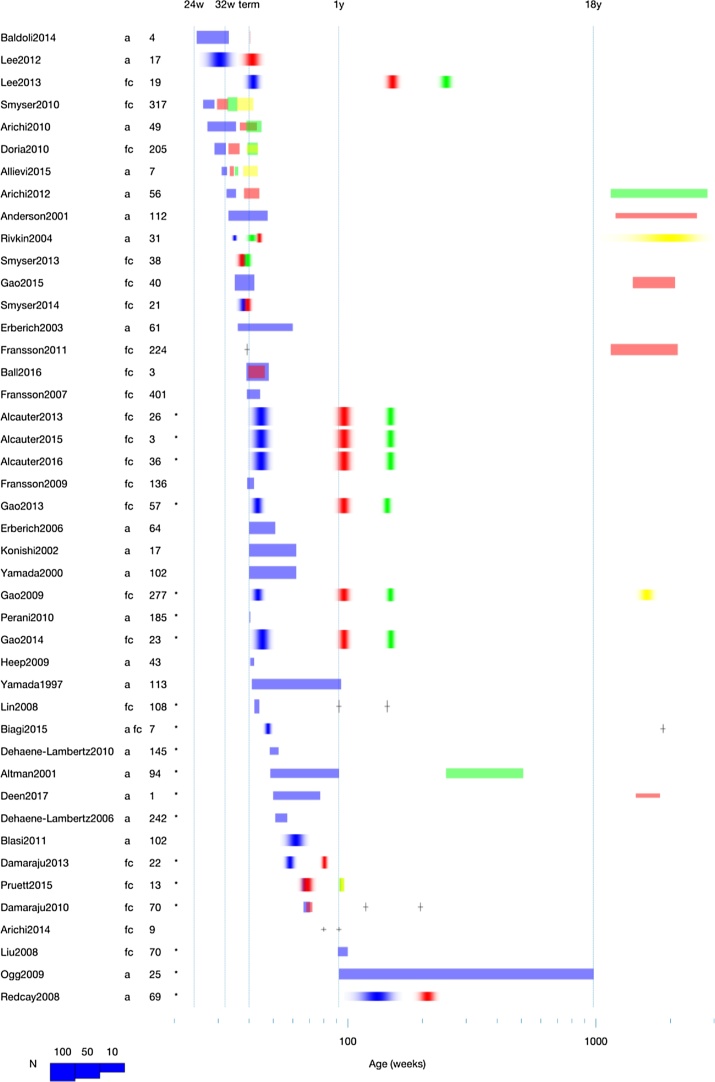
A summary of studies that have compared groups. The first column shows a reference to the study, the second whether the study was an activation (a) or functional connectivity (fc) fMRI study, the third the study’s impact as assessed by number of citations on Google Scholar in March 2017. Age on the x-axis is shown at the top for useful ages, and at the bottom in post-menstrual weeks for studies marked with an asterisk and post-birth weeks plus 40 for studies without an asterisk. Different colors label different groups and the thickness of the line the sample size. A “blurry” box with normally distributed transparency depicts the mean and standard deviation of the group’s age, and a box with even transparency shows an age range. A cross alone shows the mean, where the range or standard deviation was not found (Alcauter et al., 2013, Alcauter et al., 2014b, Alcauter et al., 2014a, Allievi et al., 2015, Altman and Bernal, 2001, Anderson et al., 2001, Arichi et al., 2014, Arichi et al., 2010, Arichi et al., 2012, Baldoli et al., 2014, Ball et al., 2016, Biagi et al., 2015, Blasi et al., 2011, Damaraju et al., 2013, Damaraju et al., 2010, Deen et al., 2017, Dehaene-Lambertz et al., 2010, Dehaene-Lambertz et al., 2006, Doria et al., 2010, Erberich et al., 2003, Erberich et al., 2006, Fransson et al., 2011, Fransson et al., 2009, Fransson et al., 2007, Gao et al., 2015, Gao et al., 2014, Gao et al., 2013, Gao et al., 2009, Heep et al., 2009, Konishi et al., 2002, Lee et al., 2012, Lee et al., 2013, Lin et al., 2008, Liu et al., 2008, Ogg et al., 2009, Perani et al., 2010, Pruett et al., 2015, Redcay et al., 2008, Rivkin et al., 2004, Smyser et al., 2014, Smyser et al., 2010, Smyser et al., 2013, Yamada et al., 1997, Yamada et al., 2000).

## 1 The changing brain

### 1.1 Scale for reproducibility

There is a growing awareness in science, and specifically in neuroimaging, psychology and neuroscience, that it is important to maximize the reproducibility of findings (Szucs et al., 2017, Poldrack et al., 2017). There are many facets to this, including increasing transparency by sharing data, analysis code and pre-registering proposals, and ensuring statistical methods are valid (Eklund et al., 2016). Critically, the power of studies must be sufficient that hypotheses can be supported or refuted. Longitudinal infant neuroimaging is no exception, and studies must be large enough that after participant dropout (or non-compliance) and quality control, there remains sufficient power. Ongoing large projects, such as the Developing Human Connectome Project (http://www.developingconnectome.org) and Baby Connectome Project (http://babyconnectomeproject.org) have tremendous potential in this respect.

### 1.2 Changing size, gyrification and shape

The head grows rapidly after birth with mean circumference increasing from 34 cm at birth to 43 cm at 6 months and then slowing, reaching 45 cm at the end of the first year and 49 cm by 5 years (De Onis et al., 2008). A similar trajectory is seen in MRI of cerebral volumes, which are on average 0.5, 0.9, 1 l at birth, 1 year and 2 years respectively, and 1.3 l in young adults (Shi et al., 2011, Peelle et al., 2012). Grey matter volume in particular grows dramatically in the first year (106%) and much less in the second year (18%) (Gilmore et al., 2012). This changing brain size has important implications for longitudinal fMRI. Before comparing brain activation or connectivity across age groups, it is important to scale brains to correct for the gross changes in size. A more subtle but important consideration of this growth is that it is not homogenous, as some brain regions have different growth trajectories to others (Gilmore et al., 2012) and show different trajectories of gyrification (Li et al., 2014). These will cause the shape of the brain to change with age. In addition to scaling, it is therefore important to warp individual brains and different ages to a common space before comparing them.

**Fig. 2:**
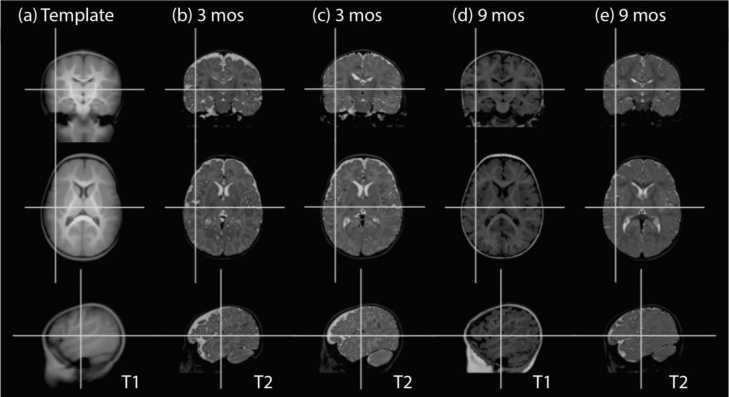
Despite changes in the brain, with appropriate algorithms robust normalization to a template (a) (Shi et al., 2011) can be obtained in different individuals at different ages, even across T1/T2 contrasts (b)–(e).

Fortunately, even in adults there are substantial differences in brain size and shape, due to sex, race, and individual differences, and rescaling and warping are already part of the warp-to-template (or normalization) algorithms built into major neuroimaging packages such as SPM, FSL, AFNI and BrainVoyager, or specialized tools such as 4D HAMMER (Shen and Davatzikos, 2004). In our experience, in infants of term age or older this registration process is robust, as although the gyri are not completely adult like they are substantially developed by the age of term birth, and sufficiently salient to provide clear features that drive image registration. Acceptable normalization can therefore be obtained by normalizing to an adult template. However, improvement in the accuracy of registration can be obtained by initially normalizing individual infants to an age-specific template (Shi et al., 2011, Altaye et al., 2008, Kuklisova-Murgasova et al., 2011) or using a procedure that iteratively creates a template from the data themselves (Ashburner, 2007) (Fig. 2). Once registered to an age-specific template, to perform longitudinal analysis it is then necessary to perform a second warping stage to register the template spaces of the two age groups. Although inter-subject registration is a frequent concern, in our experience it is unlikely to be the limiting factor on the power or spatial resolution of infant fMRI studies.

Age-specific templates that provide tissue probability maps (Kuklisova-Murgasova et al., 2011, Shi et al., 2011, Altaye et al., 2008, Makropoulos et al., 2014, Beare et al., 2016) might be of greater importance when performing segmentation, for example in order to derive timecourses from the cerebrospinal fluid (CSF) or from white matter to be used as nuisance regressors in functional connectivity analyses. Segmentation based on adult templates can result in inaccurate grey matter, white matter and CSF maps or fail because of the different properties of an infants’ brain. For instance, the amount of CSF between gray matter and the skull is typically much smaller in young infants than in adults, and the signal-to-noise ratio is lower. Additionally, in neonates and young infants, grey and white matter contrast is reversed due to physiological differences in water content, macromolecular content and myelination of the different tissue types (Rivkin, 2000, Rutherford et al., 2004). Usage of age-appropriate templates is therefore preferable when performing tissue segmentation in infants.

Finally, to preserve signal-to-noise, similar voxel sizes are typically used in infants, children and adults. Relative to the size of brain structures, resolution will therefore be lower in younger participants, which will increase partial voluming, in which different structures overlap within the same voxel. This could have consequences for both structural and functional imaging. Calculating spatial smoothness and correcting for any differences between age-groups might be necessary.

### 1.3 MRI coil selection

MRI neuroimaging uses a coil that fits snugly around the participant’s head, and sends and receives the radio-frequency waves that provide the signal. Modern MRI scanners use a phased-array coil, which has a signal-to-noise that is highest close to the coil, and drops off towards its center. In adult imaging this provides the best signal in the cortex. A consequence of the infant’s smaller brain is that much of their cortex will be further from the coil and so the signal-to-noise will be lower than in adults. Better signal might be obtained from using the smallest possible adult coils (e.g., GE or Siemens 32 channel coils). However, in practice we have encountered two difficulties in performing fMRI even with these small adult coils in infants. One is that if the infant wears typical ear defenders or headphones built into ear defenders, they do not fit into the small coils. Another is that infants have a short neck, and modern coils are shaped to have a narrowing towards where the adult neck would lie. Therefore, the infant lies at a narrow part of the coil, and is still far from many of the receiving coils. A potential solution is to use phased-array coils customized for pediatric applications (Keil et al., 2011, Deen et al., 2017, Hughes et al., 2017). In future, these could potentially be modified to provide built-in sound attenuation or audio presentation. We would recommend that the best possible coils be used in each age group to be tested. Care should then be taken, however, to ensure that differences between groups are not due to differences in sensitivity due to head shape or coil selection.

### 1.4 Hemodynamics

Neural activity is not measured directly by fMRI, but via the blood oxygen level dependent (BOLD) response. This hemodynamic response is delayed relative to neural activation, and the temporal profile of this delay is known as the hemodynamic response function (HRF). In adults, there is a peak in the HRF around 5 s after the neural response (Aguirre et al., 1998), reflecting a complex chain of events. When a brain region is active, release of noradrenaline and glutamate at the synapses act via two signaling pathways to dilate the arterioles and increase blood flow (Attwell et al., 2010). Veins swell and the increased blood flow over-compensates for the oxygen used by the brain activity, leading to a net reduction in blood deoxygenation (Buxton et al., 1998). The consequent changes in blood volume and its magnetic susceptibility lead to the BOLD response (Hoogenraad et al., 2001).

**Fig. 3:**
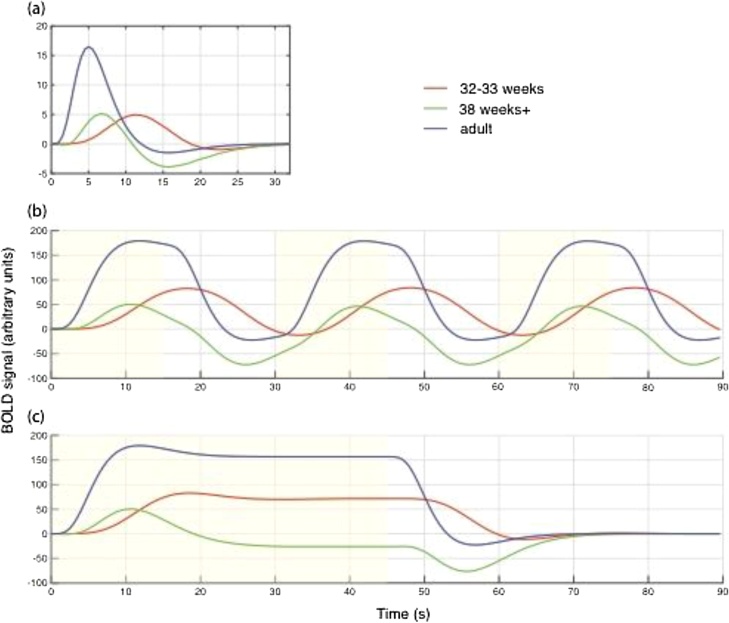
(a) The hemodynamic response (HRF) to a brief 1-s stimulation at three ages (Arichi et al., 2012). In adults the HRF is dominated by a positive peak, while at 38 weeks gestational age (GA) neonates have positive and negative peaks of similar magnitude. At 32 weeks GA the HRF is dominated by a positive peak, but it is much delayed. (b) The form of the HRF affects the power of different stimulation designs. To illustrate this, the response to a 30s-long cycle of stimulation (yellow) and rest was calculated by convolving the HRFs with a boxcar. For this design, at all three ages, there was substantial modulation of the BOLD signal through time. The signal in adults and 38 week infants was highly correlated, but at 32 weeks the signal has a different phase. (c) In contrast, for 45 s of stimulation is followed by 45 s of rest, the 38 week infants only have small peaks of modulation in the BOLD signal, and so much reduced power would be expected.

In infants, the HRF has a different size and shape (Fig. 3a) but consensus on its exact form has not yet emerged, although it has been measured using fMRI (Arichi et al., 2012, Colonnese et al., 2008, Dehaene-Lambertz et al., 2010), near-infrared spectroscopy (NIRS) (Liao et al., 2010, Telkemeyer et al., 2009, Sato et al., 2011), and optical imaging (Kozberg et al., 2013). At term age or earlier, the HRF has been shown to peak later (6–12s) in both mice (Kozberg et al., 2013, Colonnese et al., 2008) and humans (Arichi et al., 2012). The polarity of the HRF has sometimes been found to be reversed (Kozberg et al., 2013, Anderson et al., 2001, Roche-Labarbe et al., 2014) and is sometimes biphasic, with positive and then negative lobes (Kozberg et al., 2013, Arichi et al., 2012). Furthermore, the magnitude of the hemodynamic response was found to be smaller in infants (Arichi et al., 2012). These developmental changes in the HRF could reflect changes in the signaling pathways, vascular structure, or vascular physiology (Kozberg and Hillman, 2016, Harris et al., 2011).

The changes in the HRF through development have important consequences for the design and analysis of fMRI studies (Cusack et al., 2015). During the design of stimulation paradigms for fMRI, it is important to consider the effects of the HRF on power. For example, when the HRF is longer in duration, the response from rapid successive events will be more overlapping, and slower designs will be more powerful. Conversely, when the HRF is biphasic, long block designs may have less power, as the positive and negative phases will cancel (Fig. 3b). The HRF also affects the analysis of fMRI data. If an incorrect (adult) model is used for the HRF in the general linear model that typically forms the first level of analysis in fMRI studies, power will be reduced.

When comparing across age groups, paradigms and analyses should be chosen so that they do not bias the comparison. For example, if a rapid event-related design shows an effect in adults but not in infants, this might be merely due to HRF differences, and not to differences in neural processing. We would recommend designs that give equal sensitivity in the groups to be compared, and analysis strategies that are tuned to maximize power from each group. For quantitative analyses of the effects, we refer the reader to the results of simulations (Cusack et al., 2015).

Differences in HRF could also affect measures of functional connectivity. This will be particularly the case if the HRF develops at different rates and mismatches between brain regions, which would reduce the observed connectivity. Even when the HRF is similar across brain regions, its shape affects the frequency spectrum of the BOLD signal and so may affect connectivity estimates. It is noteworthy that changes in the frequency spectrum of the resting BOLD signal have been observed through development (Alcauter et al., 2014b).

### 1.5 Physiological noise

A distinction can be made between noise in MRI data due to thermal noise in the coil and noise due to physiological process in the brain, such as noise from the heartbeat and respiration, or from uncontrolled cognitive processes (Triantafyllou et al., 2005). We have found that the spectral shape of noise to be similar in infants and adults (Cusack et al., 2015). However, differences in the resting heart rate or respiratory rate, which are 2–3 times higher in newborns than in adults, might affect the signal-to-noise ratio of fMRI, particularly as better MRI coils reduce imaging noise and make physiological noise dominate at typical voxel sizes (Triantafyllou et al., 2010). Physiological recordings obtained during data fMRI acquisition from pulse-oximetry and a respiratory belt can be used to correct for physiological artifacts and are recommended.

### 1.6 Chemistry

Brain tissue changes in water concentration, macromolecular content, myelination, and vascular density in the first year, which changes its magnetic properties and affects the MRI signal (Rivkin et al., 2004). This affects the contrast of structural images, but also affects the optimal parameters for fMRI. fMRI is sensitive to the *T*2* relaxation time of the grey matter, which can more than twice of its adult value in preterm infants. In particular, the optimal echo time (*TE*) for an echo-planar fMRI acquisition depends on *T*2*. The BOLD signal (*B*) in an image is a function of *TE* and *T*2* (Deichmann et al., 2002):

$$B=k.TE*e^{-\frac{TE}{T2^{*}}}$$

where *k* is a constant of proportionality. This is the signal-to-noise for a single image, but more important for fMRI is the contrast-to-noise of the whole imaging sequence. As the number of images is inversely proportional to the *TE*, if we make the approximation that successive images are independent, this introduces an additional benefit of short acquisitions with a factor of $\frac{1}{\sqrt{TE}}$ yielding:

$$B=k.\sqrt{TE}*e^{-\frac{TE}{T2^{*}}}$$

**Fig. 4:**
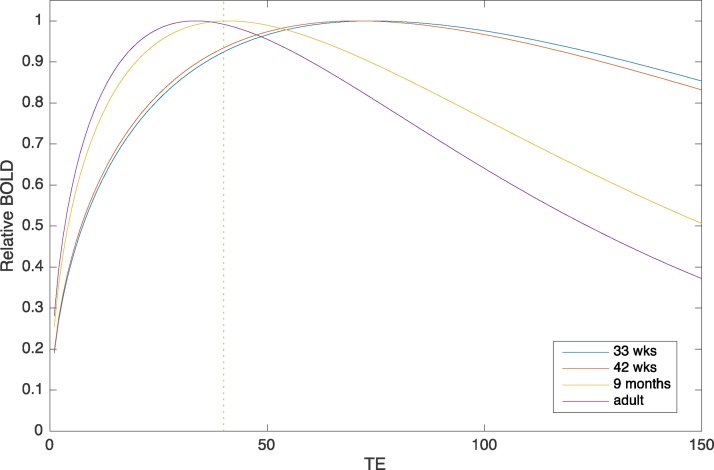
Estimated BOLD signal at 1.5 T as a function of echo time (TE, ms) relative to its maximum, at four different ages. The dotted vertical line denotes a commonly chosen echo-time for adult studies at 1.5 T.

Using this equation, estimates of BOLD contrast as a function of *TE* at four ages at 1.5 T are shown in Fig. 4 using the *T*2* averaged across medial and lateral occipital regions (149, 142, 82 and 67 ms, for 33 wks, 42 wks, 9 mos, and adults, respectively) (Rivkin et al., 2004). It can be seen that there are substantial differences in the optimal *TE* as a function of age, with longer values advantageous early in the first year. There is a further factor not considered here as it is difficult to generalize to different populations. This is that longer acquisitions give a lower sampling rate and are potentially more vulnerable to participant motion. This would suggest that the optimal acquisition will not a have a *TE* extended to the maximal value seen as optimal in the figure, but rather a compromise value that is somewhat shorter, particularly for participants that move a lot. Furthermore, the reader should be aware that at 3 T, *T*2* values (and hence the optimal *TE*s) are approximately 25% shorter.

## 2 Changing behavior

### 2.1 Infant motion

fMRI acquisitions are typically 5–20 min in duration and motion during this extended period is a substantial cause of measurement noise. Even when healthy adults are being scanned, great care is taken to minimize motion. Participants are made as comfortable as possible, repeatedly asked to remain as stationary, or even placed in a head restraint (e.g., https://caseforge.co). In recent years, awareness has increased that measures of functional connectivity are particularly disrupted by motion (Power et al., 2011, Van Dijk et al., 2011). Perniciously, the effect is not just one of increased noise, but rather a bias in the pattern of results, with longer-range connections disrupted more by movement than shorter-range connections (Ciric et al., 2016). When comparisons are made across ages there is the risk that if they move to different extents, or in a different way, it may cause an artefactual difference in the fMRI results. This has proven to be a particular problem when comparing patient groups to healthy controls, and in pediatric studies as children move more than adults in the scanner (Satterthwaite et al., 2012). This led to the conclusion that children had less long-range connectivity in their brains relative to adults, but it is not clear whether this is a true effect, or an artifact of differential motion (http://www.jonathanpower.net/2010-neuron-devo-review.html).

**Fig. 5:**
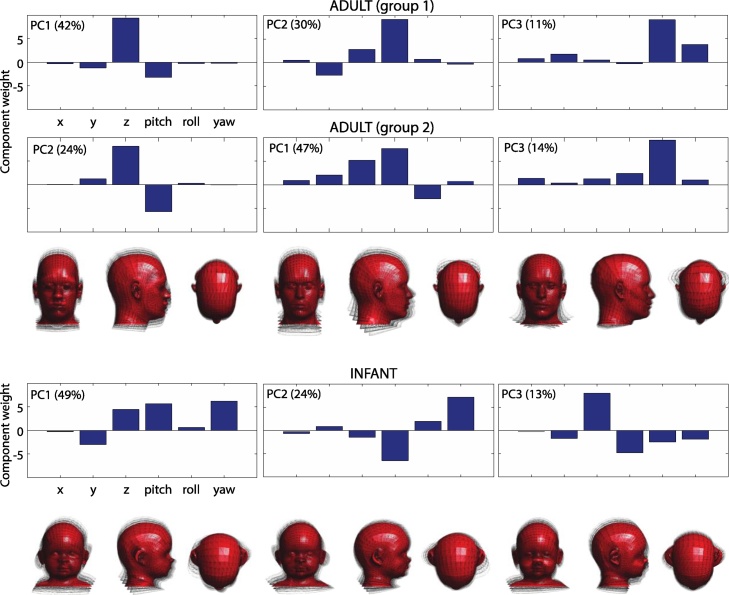
Key modes of motion for two independent groups of adults and infants, derived using principal components analysis. The top three components for each group are shown, and the percentages in brackets shows the variance explained by each component. The three-dimensional renderings visualize the three modes of motion for the adults (group 1) and the infants.

In preverbal infants, there is the additional constraint that it is not possible to communicate the request to remain still. One obvious way to reduce movement is to scan infants while they are asleep. We have shown that sleeping infants do not move more than awake adults (Cusack et al., 2017), although they do move in a different way (see Fig. 5), perhaps because of differences in head and body size, and their musculature.

**Fig. 6:**
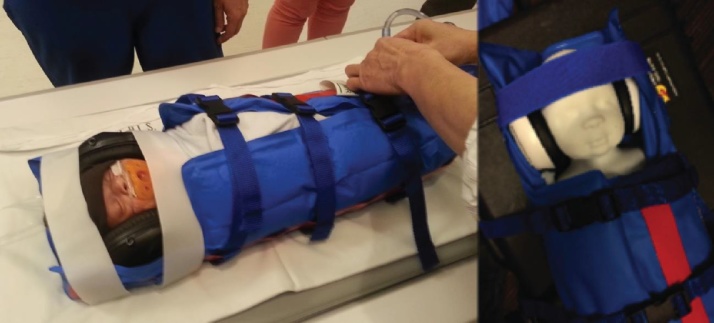
Young infants are comfortable when swaddled in an immobilizer, which reduces the need for sedation (Golan et al., 2011). The infant (left) is wearing headphones built into ear defenders.

To reduce the effect of movement during scanning, we have avoided sedation as this affects neural processing and may affect neurovascular coupling (Di Francesco et al., 2013). We recommend a number of alternative measures. First, sleeping infants move less. Second, the age at which infants are scanned should be given careful consideration. In our experience and that of other laboratories (Lee et al., 2013; Linke et al., 2017) preterm and term neonates sleep soundly in the scanner, and a high success rate can be obtained with relatively little movement. In their first year after birth, infants move more and there is a lower proportion of useable scans, but studies are still viable without sedation (Damaraju et al., 2013; Wild et al., 2017). At two years, however, infants are sufficiently mobile and willful that scanning without sedation has been proven very difficult in some studies (Lee et al., 2013) although more successful in others (Nordahl et al., 2008). But then, by four years, it is easier to reason with the participants and scanning can again be performed without sedation (Lee et al., 2013). Third, we recommend making the infants as comfortable as possible, and if they are at a young age, swaddling them. In neonates and young infants, we have also found a pneumatic infant immobilizer to be very helpful (Golan et al., 2011) (Fig. 6), while in toddlers and older children a weighted blanket can help to reduce motion and increase comfort.

During analysis, it is typical to remove data where there has been excessive movement, either by removing entire subjects, removing scanning runs, or portions of those runs (e.g., Deen et al., 2017, Smyser et al., 2010). Following realignment on the remaining data, regression can then be used to remove residual artifacts, with a nuisance regressor set of sufficient size to be effective (Ciric et al., 2016). Independent component analysis denoising has been shown to be effective in infants (Salimi-Khorshidi et al., 2014, Ball et al., 2016). Other techniques such as rapid acquisition with multiband fMRI (Setsompop et al., 2012) and multi-echo denoising (Kundu et al., 2011) may improve data quality although we are not aware of specific evaluations of their benefits for infant fMRI.

### 2.2 fMRI distortion

An issue related to movement is distortion of the echo-planar imaging (EPI) used for fMRI. Ideally, the magnetic field in the bore of the MRI scanner would be homogenous, but when a person is placed in the MRI scanner, the differences in the magnetic susceptibility of tissue, bone and air cause inhomogeneities in the field (Cusack and Papadakis, 2002). These then disrupt the imaging process and distort EPIs along the phase-encoding axis but they do not disrupt the structural images, which leads to mismatch in shape between the functional and structural images (Jezzard and Balaban, 1995, Cusack, 2003). In relatively modern scanners (e.g., Siemens Trio) the shimming process, which is typically at the start of the first fMRI scan, is effective in correcting the field distortions. However, if there is substantial head movement, or movement of a caregiver in the scanner (Biagi et al., 2015) between the calibration and EPIs, it causes distortion. One solution is to perform warp-to-template not from a structural image but from the mean EPI. If there is repeated movement through the scanning session, warping can be performed separately for each scanning session or part of it (Deen et al., 2017).

### 2.3 Sleep

It is common practice to scan infants asleep, to reduce movement. In adults, sleep has been shown to affect cognition, the brain’s response to stimuli and its functional connectivity (Tagliazucchi et al., 2012). An effect of sleep on the brain’s response to stimulation has also been found in infants (Dehaene-Lambertz et al., 2002). Care should be taken, therefore, in comparing fMRI or functional connectivity responses from sleeping infants with awake adults or children. However, even if groups of sleeping participants are compared, there may remain confounding effects due to differences in the nature of sleep with age. Infants sleep more than adults and for the first few years have one or more naps during the day (Sadeh, 2000). The typical neonatal sleep cycle is divided into periods of three to four hours throughout the normal 24-h circadian cycle. By three months of age, the infant sleep cycle matures to six-hour sleep cycles. These sleep cycles are characterized by periods of sleep during the day, with sleep consolidating to a steady nocturnal sleep cycle and a single nap during the day by one year of age (Middlemiss, 2004). Infants also have a different balance and pattern of sleep stages, with rapid-eye movement (REM) sleep dominating in early infancy (Middlemiss, 2004). Unlike adults, newborns typically enter REM or active sleep at the beginning of the sleep period; by about three months of age, the amount of REM sleep decreases and they tend to enter non-REM or quiet sleep initially (Middlemiss, 2004). All of these may confound comparisons between age groups, and should be the topic of future study.

### 2.4 Peripheral sensory changes

A potential confound in fMRI activation studies is that what appears to be a poorly developed brain function (e.g., in face detection) could actually be a result of early-stage peripheral sensory development (e.g., low visual acuity). Vision is poorly developed at birth, with lower acuity (Dobson and Teller, 1978) partly due to the early stage of eye development (Yuodelis and Hendrickson, 1986), and poorer color discrimination (Brown, 1990) than adults. Graphic examples can be seen on the website tinyeyes.com, which was created by researchers at Stanford University.

The auditory system is relatively more developed than vision at birth as many sounds can be heard in utero. The first evidence of fetal responses to sound are at 28-weeks gestational age (Birnholz and Benacerraf, 1983). However, even basic aspects of auditory processing like frequency discrimination do not fully mature until later in childhood (Litovsky, 2015). The ear canal and middle ear change in their acoustic impedance (André et al., 2012, Keefe et al., 1993). Furthermore, the challenges of delivering sounds change with age. For comfort, it is important to also provide hearing protection, through the use of earplugs (up to 30 dB), ear defenders (up to 30 dB), or MiniMuffs (at least 7 dB, Natus, Pleasanton, CA). In-ear insert headphones are attractive because they are smaller than over-the-ear circumaural headphones and could potentially allow scanning in a smaller MRI coil. However, we have found that in neonates even small-sized inserts placed by a certified audiologist can fall out. If the infant is also wearing ear defenders or MiniMuffs over the top of the ear it is then hard to determine if the inserts fell out during the scan or afterwards as the equipment was being removed. Thus, we have found that despite their larger size, circumaural headphones that also provide acoustic attenuation to be the most reliable sound presentation method in infants. The same headphones can also be used across age groups. In sum, when comparing fMRI activation results across ages, care should be taken to ensure that the results are not merely attributable to differences in sound delivery or sensory capabilities, rather than differences in brain function.

Finally, the limited communicative abilities of infants make it important to ensure that all stimuli presented are comfortable, by piloting in adults, or testing on infants outside of the scanner, taking into consideration the differences in sensory capabilities.

## 3 Challenges of interpretation

**Fig. 7:**
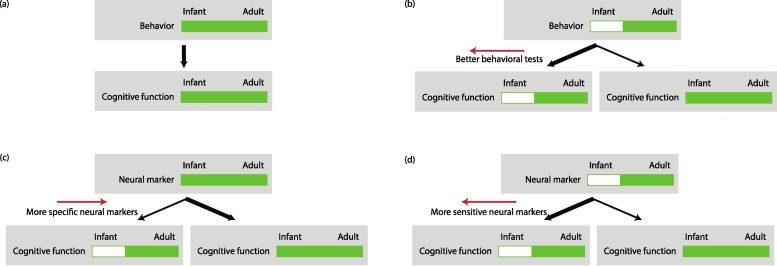
Inferences on infant cognition from behavioral and neuroimaging experiments. (a) When a behavior is observed in infants, it can be confidently inferred that the cognitive function responsible for this behavior is present. (b) A behavior is seen in adults but not in infants suggests that the cognitive function is absent in infants (heavier black arrow). However, it has often been found in developmental psychology that a behavioral test could not be performed by infants because it required some additional unintended cognitive function. So, some inferential doubt remains (lighter black arrow). Better behavioral tests will leave less room for doubt (red arrow). (c) When a neural marker is seen in adults and infants, it suggests the cognitive function is present. However, some neural markers are necessary but not sufficient for a cognitive function, and so some inferential doubt remains (lighter black arrow). Neural markers that are more specific to the presence of the cognitive function will leave less room for doubt. (d) The absence of a neural marker suggests the cognitive function is absent in infants. However, some neural measures lack sensitivity. More sensitive neural markers leave less room for doubt.

Once good data have been acquired, there is also the challenge of correctly interpreting the results. We discuss the strength of inferences that can be drawn about cognitive functioning, on the basis of behavioral or neuroimaging results. When a behavior is observed in some task that requires a given cognitive function in a group of infants, a strong inference can be made that cognitive function is present in the infants (Fig. 7a). If the behavior is seen in adults, but not in infants, then with some confidence, it suggests that the cognitive function is present in adults but absent in infants (Fig. 7b, left branch). However, there are many examples in developmental psychology where it emerged that some confounding cognitive requirement actually prevented infants from performing the behavioral task, and when a better task was designed infants were able to demonstrate the originally targeted cognitive function (Fig. 7b, right branch). For example: object permanence was thought by Piaget not to emerge until 9 months (Piaget, 1954), but has since been demonstrated at 4 months (Baillargeon, 1987); episodic memory was thought not to operate for the first year or two (Schacter and Moscovitch, 1984) but since then has been demonstrated at a few days of age (Pascalis and de Schonen, 1994); and theory-of-mind was thought to only begin after 4 years of age (Wellman et al., 2001) but then demonstrated at 7 months (Kovacs et al., 2010). Some readers will attach importance to nuance in these examples but will not doubt the principle that absence of evidence for a cognitive function in infants is not evidence for an absence.

As with many areas of science, evidence from single tasks or methods should be interpreted with caution and converging evidence from as many methods as possible sought. Neuroimaging can provide an additional, quite distinct, source of converging evidence. However, it too can have ambiguity in interpretation. In the case where a neural marker of a cognitive function is seen in infants and adults, this suggests that the cognitive function is present in infants (Fig. 7c, right branch). It is important that this neural marker is specific to the cognitive function, ideally being necessary and sufficient for it. If it is necessary, but not sufficient, then its presence may not infer the presence of the cognitive function (Fig. 7c, left branch). Unfortunately, it can be difficult to ascertain what is sufficient with full confidence, as it might be that neural components dissociate only in infancy. Finally, when a neural marker is not seen in infancy, then it suggests that the cognitive function that it is associated with is absent (Fig. 7d, left branch). However, care should be taken that the absence of evidence for the neural marker is not a result of a lack of power in the neuroimaging.

**Fig. 8:**
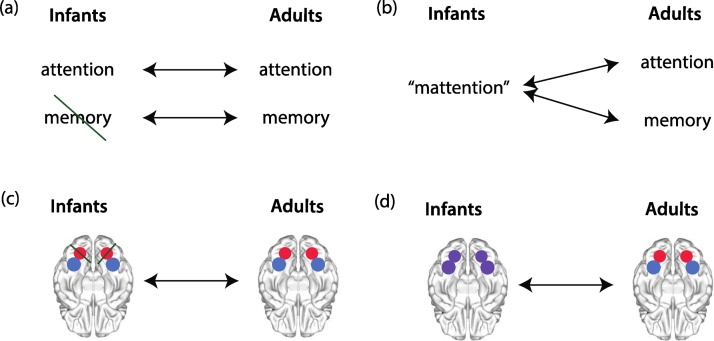
It is important to consider the appropriate way to make mappings across age groups. (a) Infant cognitive functions might be carved up in the same way as in adults, although some functions may not be yet present. In this hypothetical example, attention is present in infants and adults, but memory is only present in adults. (b) The division of cognitive functions might fundamentally different, so that attention and memory are part of a monolithic precursor in infants (here titled “mattention”). (c) Similarly, neural mechanisms might be carved up in the same way in infants and adults (red vs. blue mechanisms), although some may not be present (lines). (d) Alternatively, neural mechanisms may begin in an undifferentiated way (purple tissue) and then break into discrete functions (red and blue).

There is a further challenge in interpretation that we believe to be important, in the mapping of cognitive processes in groups of different ages. It might be that the parcellation of cognitive functions in infants mirrors that in adults (Fig. 8a), even if some cognitive functions are absent. Alternatively, the cognitive functions that dissociate in adults may not be separable in infants (Fig. 8b). Put another way, cognitive functions may not develop by appearing at a particular age, but rather by splitting from each other – much as stem cells become differentiated into different organs. A similar possibility must be considered for neural mechanisms. They may have the same structure of division in infants and adults, even if some are not yet developed (Fig. 8c). Or, a monolithic system may split into two parts during development (Fig. 8d). New atlases that parcellate the infant brain into functional modules will assist testing of these hypotheses (Shi et al., 2017).

It is common to study differences in activation or functional connectivity across ages in a-priori defined regions of interests (ROIs). These can be derived from localizer tasks or orthogonal contrasts in the study population, from the previous literature by constructing spherical ROIs around reported coordinates of activation, or from existing atlases that parcellate the brain into anatomically or functionally distinct regions [see (Poldrack 2007) for a review of ROI selection methods]. When interpreting any developmental differences in results seen, it is important to keep in mind which age group the ROIs used were derived from. It is likely, for example, that a functional parcellation of the adult brain does not accurately reflect the functional organization of an infant’s brain. Absence of activation in an ROI or reduced connectivity between ROIs in infants would therefore not necessarily suggest absence of the function ascribed to those regions in adults. Furthermore, some types of ROIs (e.g., spheres around a coordinate) might lead to greater partial volume artifacts in infants.

### 3.1 Conclusion

Longitudinal and cross-sectional infant neuroimaging with fMRI has tremendous potential, as a tool complementary to developmental psychology in the understanding of developing brain function, and as a clinical tool for characterizing abnormal development. We have described a number of methodological challenges that must be considered carefully before the design or interpretation of fMRI studies that compare groups across age, and made a number of recommendations, which are summarized in Table 1.

**Table 1:** Summary of challenges and recommendations.

CHALLENGES	RECOMMENDATIONS
Changes in Brain Size, Gyrification and Shape	
• Rapid brain growth in first year after birth, that is not homogenous across regions • Potentially inaccurate segmentation when using adult tissue probability maps • Use of the same voxel sizes across ages leads to lower spatial resolution and increased risk of partial volume effects in younger infants	• Two-step normalization to improve accuracy and correct for changes in brain size (first: age-specific template, second: group registration) • Inclusion of brain volume or head circumference as covariates in subsequent analyses • Use of age-specific templates • Calculate and correct for spatial smoothness
MRI Coil Selection	
• Reduced SNR in younger infants due to larger distance to head coil • Short neck of infants makes centering of the head in the coil difficult	• Use of smallest available coil • Calculate and correct for differences in SNR • Might necessitate using larger coils, and development of customized pediatric head coils
Hemodynamics	
• Different magnitude and shape of the HRF across development can lead to biases in task and resting state fMRI	• Modeling of the age-specific HRFs and adjustment of fMRI designs (e.g. trial duration) for equal sensitivity across ages
Physiological Noise	
• Heart rate and respiratory frequency are 2–3 times higher in newborns than in adults and might affect SNR	• Pulse-oximetry and respiratory measurements are encouraged to control for physiological artifacts
Brain Chemistry	
• Tissue changes in water concentration, macromolecular content, myelination and vascular density change magnetic properties and consequently the MRI signal	• T1 and T2-weighted structural acquisitions for optimal normalization and segmentation • Age-specific adjustment of echo-time for optimal BOLD contrast
Infant Motion	
• Young infants can not be instructed to lie still and patterns of movement change with age • Motion during structural acquisitions can lead to field distortions	• Sedation can be avoided and motion reduced by scanning during natural sleep • Use of age-specific motion constraints (swaddling and pneumatic infant immobilizers for younger infants; weighted blankets for toddlers) • Perform warp-to-template from the mean EPI
Sleep	
• Sleep cycles change drastically during early development • Hemodynamics and functional connectivity are altered by sleep	• Recording of onset of sleep and any awakenings during fMRI acquisitions by video monitoring or observation • Future studies employing simultaneous EEG recordings of sleep stages • Data acquisition during sleep also in older children and adults if comparisons with these age groups are desired
Peripheral sensory changes	
• Immature sensory processing in the periphery might be confounded with differences in brain function	• When comparing fMRI activation results across ages, care should be taken to ensure that results are not merely attributable to differences in stimulus delivery or peripheral sensory capabilities
Challenges of Interpretation	
• Absence of a behavior in young infants does not necessarily mean the corresponding cognitive function is absent (e.g. object permanence) • Presence of a neural marker of a cognitive function might be necessary but not sufficient • A-priori defined regions of interest that map unto a cognitive function in adults might not be appropriate for infants	• Interpret evidence from single tasks or methods with caution • A-priori power analysis can be used to reduce the chance that the absence of a neural marker is the result of lack of power • Development of age-specific functional parcellations for use in region-of-interest based fMRI studies

## Conflict of Interest

None.
